# Enhanced expression of ten‐eleven translocation 1 reverses gemcitabine resistance in cholangiocarcinoma accompanied by a reduction in P‐glycoprotein expression

**DOI:** 10.1002/cam4.1983

**Published:** 2019-02-19

**Authors:** Chuanxu Wang, Hua Ye, Lei Zhang, Yayu Cheng, Shifeng Xu, Ping Zhang, Zijie Zhang, Jimin Bai, Fangkang Meng, Lin Zhong, Guangjun Shi, Hao Li

**Affiliations:** ^1^ Department of General Surgery WeiFang Medical University Weifang China; ^2^ Department of Hepatobiliary Surgery The Affiliated Qingdao Municipal Hospital of Qingdao University Qingdao China; ^3^ Department of Oncology The Affiliated Hospital of Southwest Medical University, Southwest Medical University Luzhou China; ^4^ Department of Hepatobiliary Surgery The Affiliated Hospital of Jining Medical University Jining China; ^5^ Department of Gynecology The Affiliated Qingdao Center Hospital of Qingdao University Qingdao China; ^6^ Department of General Surgery Shandong Provincial Hospital Affiliated to Shandong University Ji’nan China; ^7^ Department of Gynecology The Affiliated Qingdao Municipal Hospital of Qingdao University Qingdao China; ^8^ Department of General Surgery Shanghai General Hospital, Shanghai Jiao Tong University School of Medicine Shanghai China; ^9^ Department of General Surgery Linyi People’s Hospital Linyi China; ^10^ Institut Pasteur of Shanghai, Shanghai Institutes for Biological Sciences University of Chinese Academy of Sciences Shanghai China

**Keywords:** chemotherapy resistance, cholangiocarcinoma, gemcitabine, P‐gp, TET1

## Abstract

Increasing evidence revealed that ten‐eleven translocation 1 (TET1) plays an important role in tumorigenesis and chemoresistance, but its functions in gemcitabine resistance in cholangiocarcinoma (CCA) remain unknown. This study aims to investigate the effect of TET1 on gemcitabine resistance in CCA and the possible effect on P‐glycoprotein (P‐gp) expression encoded by multidrug resistance (MDR) genes. We established two kinds of gemcitabine‐resistant CCA cell lines and confirmed its specific features. The expression of TET1 and P‐gp was evaluated in gemcitabine‐resistant CCA cells and their parental cells at mRNA and protein level by quantitative RT‐PCR and western blot analysis. After transfecting the gemcitabine‐resistant CCA cell lines with *TET1* gene or siRNA, the cell viability test was obtained to verify the effect of TET1 on the sensitivity of CCA cells to gemcitabine. And then, the possible effect of TET1 on the expression of P‐gp was examined by western blot analysis. Xenograft tumor experiment was conducted to confirm the association between TET1 and P‐gp expression under gemcitabine chemoresistance. The associations between clinical outcomes of CCA patients with chemotherapy and TET1 expression were analyzed in 82 patients. The results showed that TET1 expression was significantly decreased, and P‐gp expression was increased in gemcitabine‐resistant CCA cells. Additionally, overexpression of TET1 augmented the sensitivity of CCA cells to gemcitabine and induced the decreased expression of P‐gp in gemcitabine‐resistant CCA cells. Furthermore, multivariate Cox regression analysis indicated that TET1 expression and TNM stage were independent risk factors (*P* < 0.001) for the clinical outcomes of CCA patients with chemotherapy. Additionally, Kaplan‐Meier survival and the log‐rank test showed that decreased expression of TET1 was associated with poorer prognosis of CCA patients with chemotherapy. These findings suggest that TET1 expression reverses gemcitabine resistance in CCA accompanied by a reduction in P‐gp expression. Thus, TET1 may be a promising target to overcome chemoresistance in CCA.

## 1 INTRODUCTION

Cholangiocarcinoma (CCA) is an aggressive malignancy with features of cholangiocyte differentiation and is the most common biliary malignancy and the second most common hepatic malignancy.^1^, ^2^ The tumor originates from the ductular epithelium of the biliary tree, either within the liver (intrahepatic CCA) or more commonly from the extrahepatic bile ducts (extrahepatic CCA).^3^ Additionally, the incidence of CCA has obviously increased worldwide in recent years.^4^ However, the early stage of CCA is difficult to diagnose due to the lack of representative clinical presentation and specific tumor biomarkers, and approximately 50% of patients had missed the opportunity for surgery when they had a definite diagnosis.^5^ Meanwhile, fewer than 30% of patients with CCA could be treated with R0 resection and negative histological resection margins because of the complicated anatomical structure of CCA.

CCA is a devastating malignancy with poor prognosis and high mortality that has an overall 5‐year survival rate of <10%.^4^, ^6^ Therefore, for the patients who have unresectable tumors or positive histological resection margins, chemotherapy remains the most important and effective treatment. Gemcitabine (2´,2´‐difluorocytidinemonohydrochloride) is a pyrimidine analog that has been used widely in the treatment of various solid tumors including CCA. The National Comprehensive Cancer Network (NCCN) recommended gemcitabine as a first‐line chemotherapeutic, which revealed its significant superiority. The NCCN reported that standard treatment options, such as gemcitabine chemotherapy, were shown to have a median overall survival of 11.7 months and progression‐free survival of 8 months.^7^ Our prospective study also found that either gemcitabine alone or gemcitabine‐based regimens could extend the life of patients with unresectable CCA with an overall survival of 11 months and a progression‐free survival of 4.9 months.^8^, ^9^ Unfortunately, patients with CCA are prone to be resistant to gemcitabine, leading to recurrence of CCA. The acquired drug resistance remains the most common and primary cause clinically for chemotherapy failure and generates a lower survival rate among all CCA patients.^10^

Epigenetics refers to heritable molecular determinants of phenotype that do not alter the DNA sequence, which contains DNA methylation, histone modifications, noncoding RNAs, and chromatin structure.^11^ Aberrant DNA methylation is shown to be involved in the process of tumorigenesis, progression, and chemotherapy drug resistance because of its role in the regulation of gene expression.^12^ The ten‐eleven translocation (TET) family including three members (TET1, TET2, TET3) were identified as methylcytosine dioxygenases that can catalyze DNA demethylation. TETs induce DNA demethylation by catalyzing the conversion of 5‐methylcytosine (5‐mC) primarily to 5‐hydroxymethylcytosine (5‐hmC) in an iron and α‐ketoglutarate‐dependent manner.^13^, ^14^ Among these TET protein members, TET1 is the most concerned and plays numerous key roles in cancer process, including tumorigenesis and cancer chemoresistance.^15^ As far as tumorigenesis, it is reported that TET1 works as either oncogene or tumor suppressor by its epigenetic modification mechanisms.^16^ For instance, Huang et al reported that TET1 contributes to oncogenesis of genomic rearrangements associated with malignancy such as MLL‐rearranged leukemia.^17^ Additionally, TET1 exhibits a suppressive role in the migration and invasion of gastric cancer,^18^ lung cancer,^19^ and hepatocellular carcinoma.^20^ Recently, TET1 has been found to be involved with the chemoresistance of cancer. TET1 contributes to the progression of 5‐FU resistance through DNA demethylation of nuclear factor‐erythroid 2‐related factor 2 (Nrf2) and heme oxygenase‐1 (HO‐1) in colon cancer.^21^ Additionally, a new study suggested that TET1 promotes cisplatin resistance via demethylating the vimentin promoter in ovarian cancer.^15^ However, little is known about the potential role of TET1 in gemcitabine resistance of CCA. In the present study, we investigated the function and underlying mechanism of TET1 in regulation of the CCA response to gemcitabine.

Wattanawongdon et al established and characterized the gemcitabine‐resistant CCA cell lines, and their findings indicated that long‐term exposure of CCA cell lines to gemcitabine induced multidrug resistance (MDR).^22^ Increasing studies have reported that overexpression of P‐glycoprotein (P‐gp) plays a key role in the progress of MDR in cancer.^23^ P‐gp encoded by *ABCB1* (MDR1) gene is the best‐studied member of the ATP‐binding cassette (ABC) family transporters, which result in the extrusion of drugs and their metabolites.^24^ Furthermore, as a 170 000‐Da phosphoglycoprotein, P‐gp consists of two ATP‐binding cassettes and two transmembrane regions, was the first to be identified as a well‐known mediator of tumor drug resistance and is overexpressed in drug‐resistant tumor cells.^25^, ^26^ For instance, in cancer of the gastrointestinal tract, liver, pancreas, biliary tract, kidneys, and lung, chemoresistance was acquired by exposure to various chemotherapeutic agents or was intrinsically successively illuminated to be significantly associated with activation of the *ABCB1* gene.^27^, ^28^ Therefore, research has been carried out for decades to attempt to explore the mechanisms of regulation of *ABCB1* gene expression and have confirmed that the regulation of *ABCB1* gene is highly controlled at the chromatin level.^11^, ^29^ Additionally, epigenetic alterations are emerging as a prominent mechanism of gene regulation, including DNA methylation, histone posttranslational modifications, and noncoding RNA interaction.^29^ The molecular mechanisms responsible for the acquisition of P‐gp expression following chemotherapy have not been defined, which stimulated our interest to investigate the relationships and interactions between TET1 and P‐gp in CCA with gemcitabine resistance.

Therefore, the objectives of our study were to explore the effect of TET1 in chemotherapy outcomes of CCA patients and to investigate the possible correlations between TET1 and P‐gp in CCA with gemcitabine resistance. We found that TET1 expression was extremely decreased in CCA with gemcitabine resistance. Furthermore, overexpression of TET1 increased the sensitivity of chemoresistant CCA cells to gemcitabine and was associated with decreased expression of P‐gp in chemoresistant CCA cells. Additionally, our data showed that the expression of TET1 was significantly associated with the outcomes of CCA patients with chemotherapy. These results suggest that TET1 could provide a feasible direction for development and research about the clinical treatment of CCA.

## 2 MATERIALS AND METHODS

### 2.1 Cell culture and establishment of gemcitabine‐resistant cell lines

Two human CCA cell lines, QBC939 and HuCCT1, were used in this study. QBC939 was donated by the Type Culture Collection of the Chinese Academy of Sciences (Shanghai, China), and HuCCT1 was purchased from JCRB cell bank, National Institute of Biomedical Innovation, Health and Nutrition of Japan (#JCRB0425). HuCCT1 cells were cultured in RPMI‐1640 medium (#12633012, Gibco, Thermo Fisher Scientific, Waltham, MA), while QBC939 cells were cultured in Dulbecco's modified Eagle's medium (#12100046, Gibco); both media were supplemented with 10% fetal bovine serum (#10099141, Gibco) in a 5% CO_2 _humidified environment at 37°C.

Gemcitabine‐resistant cell lines were generated from parental QBC939 and HuCCT1 cell lines by exposure to stepwise increasing concentrations of gemcitabine over a period of 14 months. Gemcitabine was purchased from Eli Lilly Japan (Hyogo, Japan). Gemcitabine was medicated starting at 0.5 mmol/L concentrations and increased approximately twofold at each step of resistance to a final concentration of 20 mmol/L when each surviving cell colony was detected. Acquired resistant cell lines were named RG‐QBC939 and RG‐HuCCT1. Additionally, the IC50 values of the four CCA cell lines were determined by cell viability test. Both gemcitabine‐resistant CCA cell lines were grown in drug‐free medium for 2 weeks, then harvested, frozen in the liquid nitrogen, and stored at −80°C until analyzed. These drug‐resistant cells were cultured in drug‐free medium for 2 weeks before performing the experiments.

### 2.2 Colony formation assay

We further established single cell clone culture of these four cell lines and observed the differences in in vitro growth patterns. In the plate colony formation assays, 1000 log‐phase cells per well were seeded in six‐well plates and cultured at 37°C under a moist atmosphere with 5% CO2 for 2 weeks. Next, the cells were fixed with methyl alcohol and stained with 0.5% crystal violet. Finally, colonies consisting of more than 50 cells were counted under an inverted microscope. Assays were repeated three times.

### 2.3 RNA extraction and quantitative real‐time polymerase chain reaction (qRT‐PCR)

Total RNA from cells was extracted using TRIzol reagent following the manufacturer's instructions (#15596026, Invitrogen, Carlsbad, CA) and isolated using a previously described method. Total RNA（1 µg）was reversely transcribed to cDNA using Superscript Ⅲ reverse‐transcription kit according to the instructions (#10928034, Invitrogen). Real‐time PCR was performed on an ABI 7500 sequence detection system (Applied Biosystems, CA), using SYBR (Invitrogen). Glyceraldehydes‐3‐phosphate dehydrogenase (GAPDH) was used as a normalization control. The primer sequences of TET1: (forward) 5’‐CAGAACCTAAACCACCCGTG‐3’ and (reverse) 5’‐TGCTTCGTAGCGCCATTGTAA‐3’. The primer sequences of GAPDH: (forward) 5’‐GCCTCAAGATCATCAGCAATGCCT‐3’ and (reverse) 5’‐TGTGGTCATGAGTCCTTCCACGAT‐3’. The PCR cycles were performed as follows: 95℃ for 30 seconds, 40 cycles at 95℃ for 5 seconds, and 60℃ for 30 seconds. Relative changes in gene expression were determined using the 2^‐ΔΔCt ^method. The range of the obtained Ct values was 20‐28. Assays were repeated three times.

### 2.4 Plasmid and siRNA construction

Total RNA extracted from cells was transcribed into cDNA, and then, the fragment was amplified. PCR primers sequences of *TET1*: (forward) 5’‐TTAGGATCCATGTCTCGATCCCGCCATG‐3’ and (reverse) 5’‐CGGTCTAGAGACCCAATGGTTATAGG‐3’. The gene number of human *TET1* is 80312. The PCR products were digested with BamHI (#1010B, Takara, Japan) and Xbal (#1093B, Takara) and cloned into p3XFlag‐CMV‐14 vector (#E7908, Sigma) using DNA Ligation Kit (#6023, Takara). The RG‐QBC939 and RG‐HuCCT1 cells were seeded in 6‐well plates for 24 hours to reach 70%‐80% confluence and were transiently transfected with *TET1* plasmid or empty vector pcDNA3.1 for negative control using Lipofectamine 2000 (#11668019, Invitrogen) according to the manufacturer's instructions.

For transfection of siRNA, targeting cells were seeded in 6‐well plates for 24 hours to reach 30%‐50% confluence. Cells were transfected with 10‐50 nmol/L of siRNA against *TET1* by using Lipofectamine RNAiMax (#13778075, Invitrogen) based on the manufacturer's instructions. The sequence of *TET1*‐siRNA‐sense was 5’‐GGGCACAAUACAACAGAAATT‐3’, and *TET1*‐siRNA‐antisense was 5’‐UUUCUGUUGUAUUGUGCCCTT‐3’. After being transfected for 24‐72 hours, cells were harvested for subsequent treatments.

### 2.5 Cell viability test

The parental CCA cell lines (QBC939 and HuCCT1) and their gemcitabine‐resistant cell lines (RG‐QBC939 and RG‐HuCCT1) or transfected cells were seeded at 2X10^3^ cells/well in triplicate into 96‐well culture plates (Costar, Cambridge, MA) in 100 µL medium for 24 hours. In addition, diverse concentrations with 0.01‐5 mmol/L of the gemcitabine in 10 µL volumes were added to these plates. After treatment for 48 hours, 10% CCK‐8 solution (#CK04, Dojindo, Tabaru, Japan) was added to each well and incubated at 37°C in a 5% CO2 incubator for 2 hours. Absorbance was determined using a spectrophotometric plate reader at 490 nm (Bio‐Rad 680, USA). Assays were repeated three times.

### 2.6 Western blot analysis

Total proteins from the frozen tissues and cells were extracted using M‐PER Mammalian Protein Extraction Reagent (#78501, Thermo Scientific, Rockford, IL). The protein concentration was determined by using Nano drop 2000 (Thermo Fisher Scientific). Thirty micrograms of the protein samples was mixed with 5x loading dye buffer (10% SDS glycerol, 1 mol/L Tris‐Cl, pH 6.8) (#P0015, Beyotime, Shanghai, China), heated at 90°C for 10 minutes, and proteins were separated by electrophoresis in 10% SDS‐polyacrylamide gel (Sigma‐Aldrich, St, Louis, MO). Next, a gel and membrane sandwich was made in a tank, and protein was wet transferred onto 0.45 μm PVDF membranes (#FFP26, Beyotime) by using a transfer buffer (#P0021A, Beyotime) at constant current 300 mA for 2 hours. The membranes were blocked with 5% fat‐free milk in Tris‐buffered saline and 0.1% Tween 20 solution at room temperature, and then incubated with primary antibodies against TET1 (#ab191698, Abcam, Cambridge, UK, 1:1000), P‐gp (#ab170904, Abcam, 1:1000), E‐cadherin (#ab1416, Abcam, 1:1000), and β‐actin (#ab8226, Abcam, 1:1000) diluted in TBST containing 5% skim milk powder at 4°C overnight. Thereafter, the membranes were incubated with secondary antibody (#ab98624, Abcam, 1:5000) diluted in TBST containing 5% skim milk powder at room temperature for 2 hours. Images of proteins were displayed using Western Chemiluminescence HRP Substrate (#WBKLS0500, Millipore, USA), and the blots were exposed to X‐ray film. The Quantity One software (Bio‐Rad, CA) was used to quantitate the gray scale of each protein strip. Assays were repeated three times.

### 2.7 Mouse and tumor injections

Twenty 6‐week‐old male nude mice were raised in a specific pathogen‐free environment and temperature‐controlled room at 22°C. The mice were divided equally and randomly into four groups. QBC939 and RG‐QBC939 cells (1 × 10^6^ in 20 μL PBS) were pairwise subcutaneously inoculated into the backs of four groups of nude mice equally. Tumor volumes were measured every week with a caliper using the following formula: V (mm^3^) = d^2^×D/2, where d and D represent the minor tumor axis and the major tumor axis. The two groups of mice inoculated with QBC939 and RG‐QBC939 cells were treated with 400 mg/kg gemcitabine at the first day through intraperitoneal injection when tumors reached a minimum size of 150 mm^3^ and treated with 200 mg/kg gemcitabine at the eighth day and fifteenth day, respectively. Moreover, the other two groups of mice without treatment of gemcitabine were raised under the same conditions as control. Mice were killed after 28 days (4 weeks) and necropsied, and tumor weights were measured. All animals received humane care per the criteria outlined in the “Guide for the Care and Use of Laboratory Animals” issued by the National Institutes of Health (NIH publication 86‐23 revised 1985).

### 2.8 Immunohistochemistry (IHC)

The tumor tissues were collected from mice as described and fixed in 4% formaldehyde overnight at 4℃, paraffin embedded, and sectioned at 4 µm thickness. Paraffin‐embedded sections were dewaxed through xylene cycles and rehydrated through a graded series of alcohol, followed by antigen retrieval with 0.01 M sodium citrate buffer (pH 6.0) in a microwave oven for 10 minutes. The sections were then treated with 3% hydrogen peroxide at room temperature for 10 minutes to block endogenous peroxidase activity. And then, samples were blocked with normal goat serum at room temperature for 15 minutes. After blocking, the sections were incubated with a rabbit monoclonal anti‐TET1 (#ab191698, Abcam, 1:300), anti‐P‐gp (#ab103477, Abcam, 1:50), or anti‐Ki67 (#ab15580, Abcam, 1:500) antibody diluted in PBS at 4°C overnight. Sections were rinsed with PBS and incubated with goat anti‐rabbit HRP‐conjugated secondary antibody (#ab205718, Abcam) at room temperature for 30 minutes and diaminobenzidine (DAB) as substrate (#ab64264, HRP/DAB IHC Detection kit, Abcam). Finally, slides were counterstained with hematoxylin and mounted for light microscopy analysis. Both staining density and intensity were scored for each slide, which were calculated using semiquantitative scoring method. Particularly, staining scores were assigned according to the percentage of positive tumor cells: 1 (up to 25% positive cells), 2 (25%‐50% positive cells), 3 (50%‐75% positive cells), and 4 (more than 75% positive cells). Additionally, the intensity scores ranged between 0 and 3—0 (no staining), 1 (week staining), 2 (moderate staining), and 3 (strong staining). A final score between 0 and 12 was calculated by multiplying the staining score with the intensity score. A score of 0‐6 indicates low expression, whereas the score of 7‐12 signals high expression.

### 2.9 Patients and specimens

CCA specimens were obtained from 82 CCA patients without distant metastasis who underwent surgery in Shanghai First People's Hospital, Shanghai, China, between 2008 and 2014. The diagnoses were all confirmed by histopathologic examination. All these patients received conventional chemotherapy with gemcitabine alone or gemcitabine‐based regimens because their tumors were accompanied with advanced tumor stage, R1/R2 surgical margins, or lymph node metastasis. Written informed consent was obtained from each patient. The study protocol was approved by the ethics committee of our institution.

### 2.10 Statistical analysis

The results are expressed as the mean ± standard deviation (SD) and analyzed using SPSS22.0 software for windows (Chicago, IL). Before analyzing the statistical significance of differences, we tested the normal distribution of data by using the Shapiro‐Wilk test. Differences between the two groups were assessed using a two‐tailed Student's *t*‐test for normal distributions and Mann‐Whitney *U*‐tests for nonparametric distributions. Additionally, when comparing three or more groups, statistical significance was calculated by one‐way ANOVAs for normal distributions and Kruskal‐Wallis tests for nonparametric distributions. For qRT‐PCR and western blot assay, quantitative data between groups were compared using Mann‐Whitney *U*‐test. Other quantitative data analysis was performed using two‐tailed Student's *t*‐test. Overall survival and disease‐free survival were estimated using the Kaplan‐Meier method, and the difference in survival was evaluated using the log‐rank test. Differences with a value of *P* < 0.05 were considered statistically significant.

## 3 RESULTS

### 3.1 Characterization of gemcitabine‐resistant CCA cell lines

**Figure 1:**
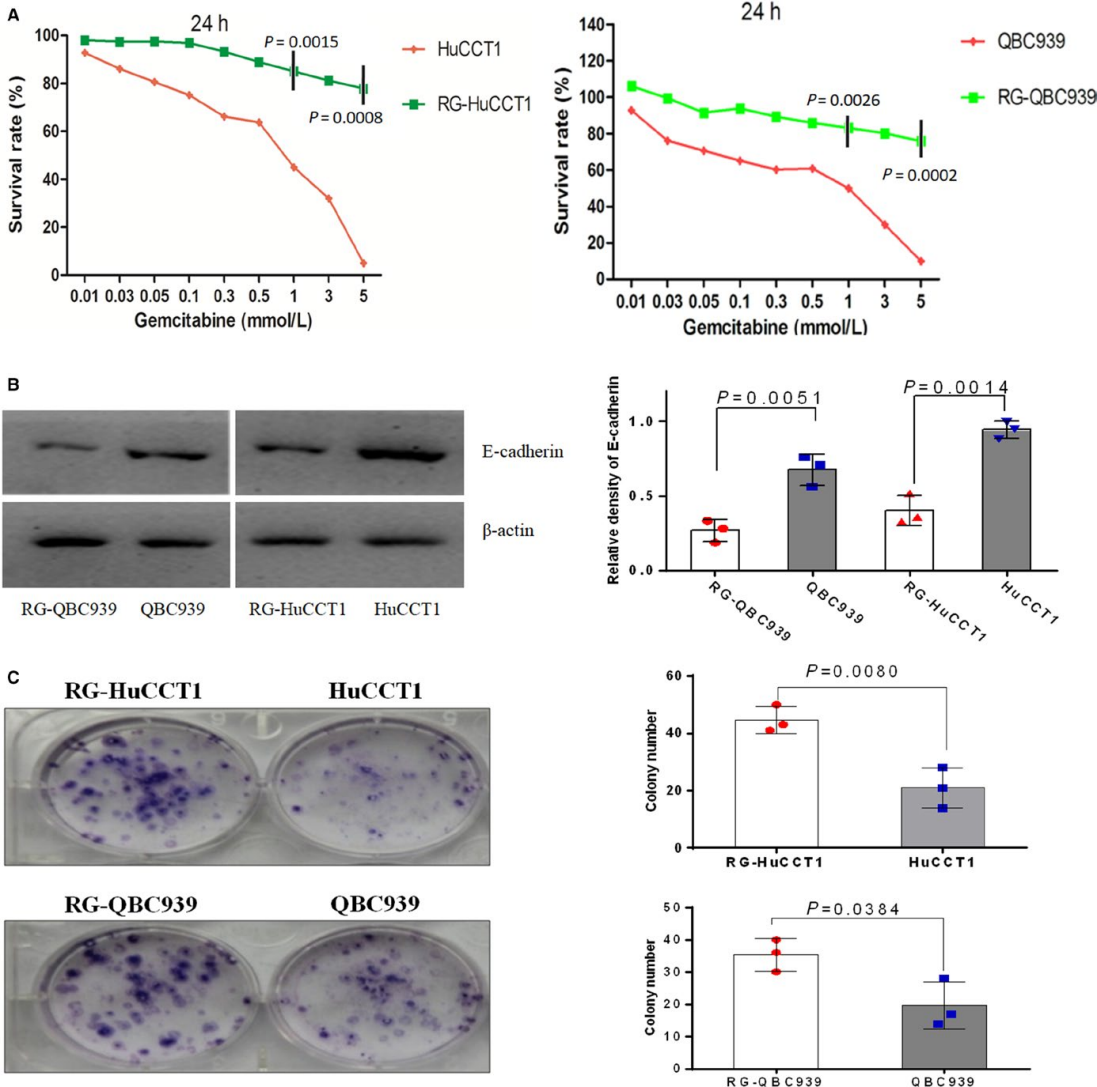
Characterization of gemcitabine‐resistant CCA cell lines RG‐HuCCT1 and RG‐QBC939. (A) HuCCT1 and QBC939 cells were incubated with various concentrations of gemcitabine. The results of cell viability assay showed that proliferation ability of RG‐HuCCT1 and RG‐QBC939 cells was higher than that of their parental cells after treating with gemcitabine. The experiments were performed in triplicate wells three times. The *P*‐values represent the results of two‐tailed Student's *t*‐test for two groups. (B) The expression of E‐cadherin in CCA cells was determined by western blot analysis. The loss of E‐cadherin expression was observed in gemcitabine‐resistant cells compared with their parental cells, which means gemcitabine‐resistant CCA cell lines may possess the stronger capacities of migration and invasion. The experiments were performed three times and each dot indicates one technical repeat. The *P*‐values represent the results of Mann‐Whitney U‐test for two groups. (C) Colony formation was performed in Petri dishes untreated for cell adhesion. At 14 days after seeding, colonies were stained with crystal violet and counted. Results showed that gemcitabine‐resistant cells were more likely to form colony units than their parental cells. The experiments were performed in triplicate wells three times. Dots represent data from cells in triplicate well under the same treatment. The *P*‐values represent the results of two‐tailed Student's *t*‐test for two groups. Data were mean ±SD

The two gemcitabine‐resistant CCA cell lines were established as previously described. The sensitivities of these cells to gemcitabine were determined by CCK‐8 assay, and the results revealed that RG‐QBC939 and RG‐HuCCT1 cell lines are significantly more resistant to gemcitabine treatments than their parental cell lines (Figure 1A, *P* < 0.001). The IC50 values of these four CCA cell lines QBC939 and RG‐QBC939, HuCCT1 and RG‐HuCCT1 were 1.2 and 14.4 mmol/L, 0.8 and 12 mmol/L, respectively. Additionally, the resistance index (RI) for RG‐QBC939 and RG‐HuCCT1 cell lines and their parental cell lines, respectively, is 12 and 15. Next, the expression of E‐cadherin was determined by western blot analysis to measure invasiveness of gemcitabine‐resistant cells. The loss of E‐cadherin expression that is the characteristic of EMT process was observed in gemcitabine‐resistant cells compared with their parental cells, which means gemcitabine‐resistant CCA cell lines may possess the stronger capacities of migration and invasion (Figure 1B, *P* = 0.0051, 0.0014 respectively). Additionally, after performing single cell clone culture of acquired variant gemcitabine‐resistant cells and their parental cells for 14 days, we found that gemcitabine‐resistant cells are more likely to form colony units than their parental cells, which means RG‐HuCCT1 and RG‐QBC939 cell lines represent a stronger proliferative ability (Figure 1C, *P* = 0.0080, 0.0384, respectively). In sum, these morphological differences indicate that this acquired gemcitabine resistance by long‐term exposure of CCA cell lines to gemcitabine enhanced their invasiveness.

### 3.2 TET1 expression at the mRNA and protein level is decreased in gemcitabine‐resistant CCA cells

**Figure 2:**
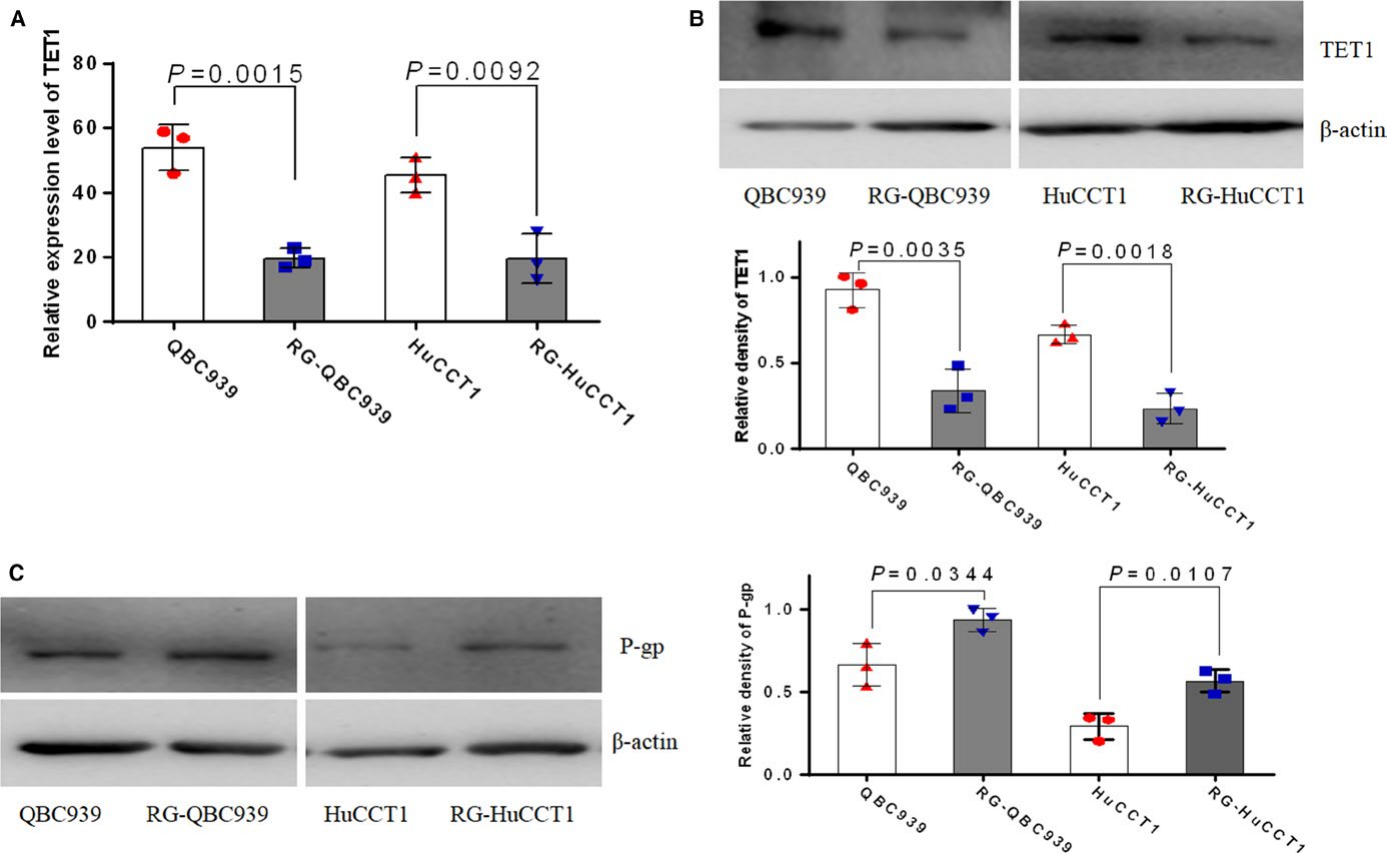
Expression of TET1 and P‐gp in gemcitabine‐resistant CCA cell lines. (A) Quantitative RT‐PCR analysis resulted in regarding the expression of *TET1* mRNA in RG‐HuCCT1 and RG‐QBC939 cells and their parental cells. The expression of *TET1* mRNA was decreased in gemcitabine‐resistant CCA cells. Dots represent data from triplicate of pipetting for measurement of qPCR. (B) Western blot analysis of TET1 expression in RG‐HuCCT1 and RG‐QBC939 cells and their parental cells. The expression of TET1 protein was decreased in gemcitabine‐resistant CCA cells. The experiments were performed three times and each dot indicates one technical repeat. (C) Western blot analysis of P‐gp expression in RG‐HuCCT1 and RG‐QBC939 cells and their parental cells. The expression of P‐gp was increased in gemcitabine‐resistant CCA cells. The experiments were performed three times and each dot indicates one technical repeat. The *P*‐values represent the results of Mann‐Whitney U‐test for two groups. Data were mean ±SD

The gene expression levels of *TET1* at the mRNA level were determined by the quantitative real‐time PCR analysis of two gemcitabine‐resistant CCA cell lines and their parental cell lines. The expression levels of *TET1* mRNA in both gemcitabine‐resistant cells were significantly lower than that of their parental cells (Figure 2A, *P* = 0.0015, 0.0092, respectively). Consistently, compared to the parental cell lines, the TET1 protein expression evaluated by western blot analysis was significantly lower in gemcitabine‐resistant cell lines RG‐QBC939 (*P* = 0.0035) and RG‐HuCCT1 (*P* = 0.0018) (Figure 2B).

### 3.3 Overexpression of P‐gp protein in gemcitabine‐resistant CCA cells

We evaluated the P‐gp protein expression in these two gemcitabine‐resistant cell lines and their parental cell lines using western blot analysis. The results showed that the expression of P‐gp in RG‐QBC939 and RG‐HuCCT1 cell lines was significantly higher than that in their parental cells (Figure 2C, *P* = 0.0344, 0.0107, respectively).

### 3.4 TET1 augments the sensitivity of gemcitabine in gemcitabine‐resistant CCA cells

**Figure 3:**
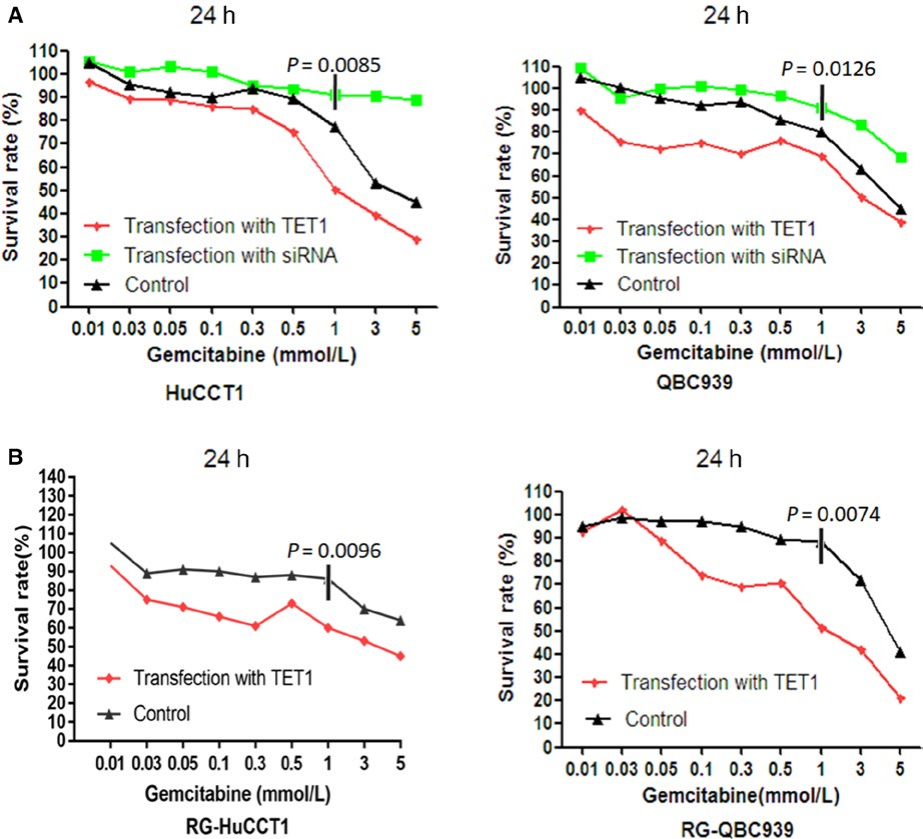
TET1 enhanced the sensitivities of CCA cell lines and gemcitabine‐resistant CCA cell lines to gemcitabine. (A) HuCCT1 and QBC939 cells upon transfection of *TET1* gene, siRNA‐*TET1*, or untreated were incubated with various concentrations of gemcitabine, respectively. The results of cell viability assay showed that overexpression of TET1 increased the responses of CCA cells to gemcitabine, while knockdown of TET1 decreased the responses of CCA cells to gemcitabine. The *P*‐values represent the results of one‐way ANOVA for three groups. (B) RG‐HuCCT1 and RG‐QBC939 cells upon transfection of *TET1* gene or untreated were incubated with various concentrations of gemcitabine, respectively. The results of cell viability assay showed that overexpression of TET1 enhanced the sensitivities of gemcitabine‐resistant CCA cells to gemcitabine, which means TET1 contributed to the reversion of chemoresistance in CCA cells. The experiments were performed in triplicate wells three times. The *P*‐values represent the results of two‐tailed Student's *t*‐test for two groups

To investigate the presumptive roles of TET1 on the sensitivity of gemcitabine in gemcitabine‐resistant cells and their parental cell lines, RG‐QBC939 and RG‐HuCCT1 cells, QBC939 and HuCCT1 that were transfected with *TET1* gene or siRNA were used to perform a cell viability test. *TET1* transfected in QBC939 and HuCCT1 cells significantly increased the mortality of cells than siRNA‐*TET1* transfected in QBC939 and HuCCT1 cells by exposure to gemcitabine (Figure 3A, *P* = 0.0085, 0.0126, respectively). The *TET1* gene‐transfected gemcitabine‐resistant cells showed significantly lower cell viability than untreated gemcitabine‐resistant cells by exposure to gemcitabine (Figure 3B, *P* = 0.0096, 0.0074, respectively). In other words, the results revealed that overexpression of TET1 in gemcitabine‐resistant cells will augment the sensitivity of chemotherapy and reverse the chemoresistance. Low expression of TET1 in gemcitabine‐resistant cells will reduce the sensitivity of chemotherapy and contribute to the chemoresistance.

### 3.5 P‐gp expression was inversely correlated with TET1 expression and immunohistochemical analysis in xenograft tumor tissues

**Figure 4:**
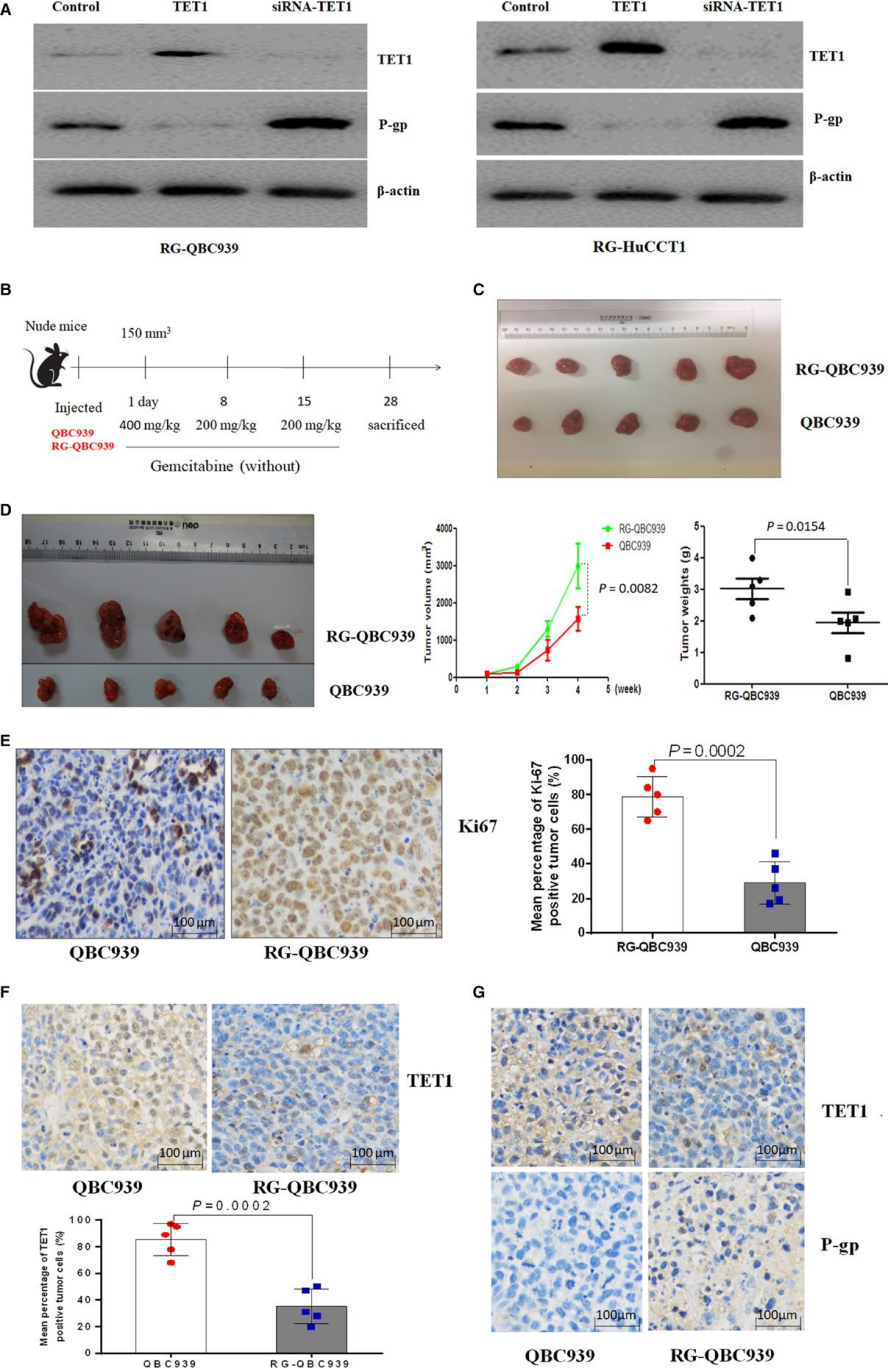
The association between P‐gp expression and TET1 and immunohistochemical analysis in xenograft tumor tissues. (A) Western blot analysis of TET1 and P‐gp expression in RG‐QBC939 cells transfected with *TET1* gene or siRNA‐*TET1*. The results showed that compared with untreated cells, p‐gp expression remarkably decreased in cells with higher expression of TET1 and increased in cells with lower expression of TET1. The experiments were performed three times. (B) Nude mice were raised, divided equally and randomly into four groups, and subcutaneously inoculated with QBC939 and RG‐QBC939 in the backs. Two groups of mice were treated with 400 mg/kg gemcitabine at the first day when tumors reached a maximum size of 150 mm^3^ and treated with 200 mg/kg gemcitabine at the eighth day and fifteenth day. The other two groups of mice were not treated with gemcitabine as control groups. Each mouse was weighed weekly, and mice were killed after 28 days (4 weeks) and necropsied. Tumor volume was monitored every week, and tumor weights were measured at the fourth week after mice were killed. (C) Tumors from the mice without treatment of chemotherapy had no significant differences in volume and weights between two groups. (D) After mice‐implanted CCA cells were treated with a conventional course of chemotherapy for 4 weeks, tumors, including the average tumor volume weekly and tumor weights derived from RG‐QBC939 cells, appeared larger than those in the other group. (E) Tumor tissues from QBC939 and RG‐QBC939 cells were immunohistochemically stained for Ki67. Ki67 expression was significantly higher in tumor tissues from mice with implanted RG‐QBC939 than that with implanted QBC939. Dots represent IHC score from 10 mice tumor tissues. The magnification is 200 times. (F) The expression of TET1 in tumors was immunohistochemically analyzed and the results showed that TET1 was expressed lowly in tumors derived from RG‐QBC939 cells. Dots represent IHC score from 10 mice tumor tissues. The magnification is 200 times. (G) Correlations between TET1 and P‐gp expression in cholangiocarcinoma tissues. The results suggested that expression of TET1 inversely correlated with P‐gp expression. The magnification is 200 times. The *P*‐values represent the results of two‐tailed Student's *t*‐test for two groups. Data were mean ±SD

P‐gp expression was assessed by western blot analysis in RG‐QBC939 cells and RG‐HuCCT1 cells transfected *TET1* gene or siRNA‐*TET1* and untreated cells as control groups. We found that compared with untreated cells, p‐gp protein expression remarkably decreased in cells with higher expression of TET1 and increased in cells with lower expression of TET1. In other words, the overexpression of TET1 was associated with decreased expression of P‐gp, whereas knockdown of TET1 was associated with increased expression of P‐gp (Figure 4A).

Furthermore, we implanted QBC939 cells and RG‐QBC939 cells into nude mice to study the characteristics of gemcitabine‐resistant CCA cells and the association between TET1 and P‐gp expression under gemcitabine chemoresistance in vivo. After mice‐implanted CCA cells were treated with a conventional course of chemotherapy for 4 weeks (Figure 4B), tumors, including the average tumor volume weekly and tumor weights derived from RG‐QBC939 cells, appeared larger than those in the other group (Figure 4D, *P* = 0.0082, 0.0154, respectively). Meanwhile, tumors from the mice without treatment of chemotherapy had no significant differences in volume and weights between two groups (Figure 4C). These results indicated that existence of difference in tumor size was due to gemcitabine resistance.

Next, further immunohistochemical analyses were carried out in tumors from mice treated with a course of gemcitabine. To further verify the proliferation characteristic of RG‐QBC939 cells in tumors from treated mice, Ki67, a crucial proliferation marker, was stained in xenograft tumor tissues. Ki67 expression was significantly higher in tumor tissues from mice with implanted RG‐QBC939 than that with implanted QBC939 after treating with gemcitabine (Figure 4E, *P* = 0.0002). In aggregate, gemcitabine‐resistant cells possessed stronger proliferation capacities under the same chemotherapeutic treatment. Additionally, TET1 and P‐gp were stained on xenograft specimens. Compared with that derived from QBC939 cells, the expression levels of TET1 protein were decreased in tumor tissues derived from RG‐QBC939 cells (Figure 4F, *P* = 0.0002). Afterward, in the same tumor tissues derived from RG‐QBC939 cells, expression of TET1 inversely correlated with P‐gp expression (Figure 4G). Overall, the results showed that P‐gp expression is significantly associated with TET1, and that TET1 possibly induces a reduction in the expression of P‐gp.

### 3.6 Downregulation of TET1 expression was associated with poorer survival of CCA patients with chemotherapy

**Figure 5:**
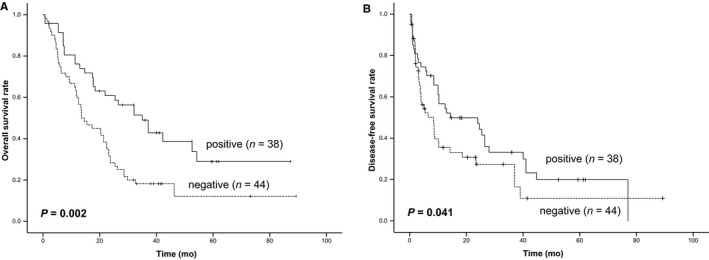
Downregulation of TET1 expression was associated with poorer survival of CCA patients with chemotherapy. (A) Kaplan‐Meier analysis of the correlation between TET1 expression and disease‐free survival in CCA patients (n = 82). (B) Kaplan‐Meier analysis of the correlation between TET1 expression and overall survival in CCA patients (n = 82). Patients with decreased expression of TET1 had poorer disease‐free survival and overall survival. The *P*‐values represent the results of Kaplan‐Meier analysis and log‐rank tests for two groups

To investigate the clinical sense of TET1 on the chemotherapeutic effects of CCA patients, survival analysis in 82 CCA patients with chemotherapy was performed. The median age of the CCA patients at surgery was 48 years (range 32‐65 years), and there were males and females. The mean follow‐up period was 28 months (range 2‐89 months). The expression levels of the TET1 protein in CCA tissues were determined by IHC analysis. The clinicopathological variables of the patients were subjected to Cox regression analysis. For disease‐free survival (DFS), TET1 expression (HR=2.364, 95%CI 1.434‐3.750; *P* = 0.004), microvascular involvement (HR=2.105, 95%CI 0.831‐4.135; *P* = 0.041), and TNM stage (HR=1.754, 95%CI 1.237‐2.378; *P* = 0.004) were found to be independent risk factors (Table 1). Additionally, for overall survival (OS), TET1 expression (HR=2.712, 95%CI 1.627‐5.312; *P* < 0.001), microvascular involvement (HR=3.062, 95%CI 1.192‐4.936; *P* = 0.026), TNM stage (HR=1.987, 95%CI 1.576‐2.859; *P* < 0.001), lymph node metastasis (HR=2.854, 95%CI 1.051‐7.972; *P* = 0.047), and CA19‐9 expression (HR=1.973, 95%CI 1.374‐3.806; *P* = 0.025) were found to be independent risk factors (Table 1). Furthermore, in multivariate Cox regression analysis, we excluded lymph node metastasis to avoid collinearity (tolerance>0.1). The results showed that TET1 expression and TNM stage were significant factors for disease‐free survival (HR=3.162, 95% CI 1.496‐4.253; *P* = 0.003 and HR=2.953, 95%CI 1.788‐4.930; *P* < 0.001) and overall survival (HR=3.760, 95%CI 2.072‐6.843; *P* < 0.001 and HR=2.368, 95%CI 1.519‐3.584; *P* < 0.001) (Table 1). Moreover, Kaplan‐Meier survival and the log‐rank test were performed to analyze TET1 expression for OS and DFS. There were significant differences in DFS and OS among CCA patients with negative and positive expression of TET1 protein. Patients with positive expression of TET1 in tumors showed longer OS and DFS than patients with negative TET1 expression. (*P* = 0.002 and 0.041, respectively) (Figure 5A,B). These findings displayed that decreased expression of TET1 was associated with poorer prognosis of CCA patients with chemotherapy.

**Table 1:** Univariate and multivariate analysis of factors associated with disease‐free survival (DFS) and overall survival (OR) of cholangiocarcinoma patients

Variables	Hazard ratio (95% CI) (DFS)	*P* value (DFS)	Hazard ratio (95% CI) (OS)	*P* value (OS)
Univariate analysis				
TET1 (low vs high)	2.364 (1.434‐3.750)	0.004	2.712 (1.627‐5.312)	<0.001
Gender (male vs female)	0.731 (0.438‐1.461)	0.506	1.362 (0.742‐2.310)	0.439
Age (>50 vs ≤50)	0.876 (0.907‐1.253)	0.053	0.982 (0.968‐1.485)	0.438
HBV (positive vs negative)	0.721 (0.501‐1.177)	0.289	0.764 (0.625‐1.201)	0.232
Tumor size (>5 cm vs ≤5 cm)	1.052 (0.979‐1.156)	0.265	1.075 (0.998‐1.325)	0.370
Liver cirrhosis (yes vs no)	0.698 (0.374‐1.385)	0.205	0.874 (0.425‐1.674)	0.427
Microvascular involvement (positive vs negative)	2.105 (0.831‐4.135)	0.041	3.062 (1.192‐4.936)	0.026
Differentiation (Poorly vs well + moderately)	1.536 (0.858‐3.784)	0.097	1.258 (0.575‐2.641)	0.378
TNM stage (III‐IV vs I‐II)	1.754 (1.237‐2.378)	0.004	1.987 (1.576‐2.859)	<0.001
Lymph node metastasis (yes vs no)	1.494 (0.563‐4.729)	0.102	2.854 (1.051‐7.972)	0.047
CA19‐9 (>100 vs ≤100)	1.305 (0.647‐2.212)	0.248	1.973 (1.374‐3.806)	0.025
AFP (>20 ng/ml vs ≤20 ng/ml)	1.036 (0.999‐1.251)	0.794	1.502 (0.954‐1.946)	0.627
Multivariate analysis				
TET1 (low vs high)	3.162 (1.496‐4.253)	0.003	3.760 (2.072‐6.843)	<0.001
TNM stage (III‐IV vs I‐II)	2.953 (1.788‐4.930)	<0.001	2.368 (1.519‐3.584)	<0.001

*P* < 0.05 was considered statistically significant.

## 4 DISCUSSION

Some clinical studies, even including one randomized controlled trial have suggested that gemcitabine‐based chemotherapy appeared to be extremely effective and well tolerated 30, but the incidence of chemoresistance has increased in recent years, which was responsible for the reduced survival of patients with CCA. From the view of the EMT process of the gemcitabine‐resistant CCA cell lines, low expression of E‐cadherin was observed in gemcitabine‐resistant cells compared with their parental cells; chemoresistant CCA cells exhibited significantly enhanced invasiveness. At the same time, colony formation assay showed that chemoresistant CCA cells possessed a stronger proliferative ability. Therefore, it is urgent to investigate the molecular mechanisms underlying gemcitabine resistance in CCA patients and then figure out feasible strategies to improve the efficacy of chemotherapy and the clinical prognosis of CCA patients. In this study, we first explored the roles of TET1 on gemcitabine sensitivity and the association between the expression of TET1 and P‐gp in CCA with chemoresistance.

In the present study, we primarily focused on the role of TET1 in gemcitabine‐resistance of CCA by in vitro and in vivo experiments. First, the expression of *TET1* mRNA and protein in CCA cells was detected and compared to the parental cell lines; the results showed that TET1 expression is decreased in gemcitabine‐resistant CCA cells. Consistently, immunohistochemical analysis of TET1 on xenograft specimens revealed that the expression levels of TET1 protein were decreased in tumor tissues derived from RG‐QBC939 cells compared with that derived from QBC939 cells. Second, we transfected two gemcitabine‐resistant cell lines with *TET1* gene and siRNA‐*TET1* and performed the cell viability test. The results showed that overexpression of TET1 could increase the sensitivity of CCA cells to gemcitabine, while loss of TET1 in CCA cells could result in gemcitabine resistance. Finally, we performed survival analysis in 84 CCA patients with chemotherapy to investigate the clinical significance of TET1, and the results suggested that decreased expression of TET1 was associated with poorer prognosis of CCA patients with chemotherapy. These findings suggested that TET1 could be a potential target for CCA with chemoresistance.

TET1, as a crucial DNA demethylation enzyme, plays vital roles in DNA demethylation regulation by converting 5‐methylcytosine to 5‐hydroxymethylcytosine.^13^ Additionally, since the TET protein family, especially TET1, was discovered in 2009, its function and regulatory mechanism in cancer have been widely investigated. Additionally, those previous studies have reported that TET1 plays roles in not only tumor suppression but also promotion during tumorigenesis and progression by regulating gene expression in a multilayered manner, including acting as transcription factor and regulating the 5hmC level.^16^, ^31^ Furthermore, in terms of chemoresistance in cancer, only a few studies have focused on the role of TET1 and its potential mechanisms. Xi Han et al found that TET1 expression resulted in resistance to cisplatin, and that one of the targets of TET1 action was vimentin involved in partial epithelial‐to‐mesenchymal (EMT) in ovarian cancer.^15^ Wei Wang et al provided evidence that decreased expression of TET1 may strengthen the sensitivity of oral squamous cell carcinoma stem cells to chemotherapeutics by stimulating MGMT promoter methylation to suppress MGMT mRNA expression.^32^ However, research revealed that gemcitabine‐resistant CCA cells displayed not only MDR but also enhanced invasiveness.^23^ Moreover, the gemcitabine‐resistant CCA cell lines we established at the beginning also exerted advanced malignance, in conformance, like the active EMT process and form more colony units. Additionally, *ABCB1* was highlighted as a fundamental factor contributing to MDR and overexpression of P‐gp is significantly associated with the acquisition of MDR as well as the reduced sensitivity to chemotherapy in many cancers.^33^, ^34^ Therefore, we investigated the association between TET1 and P‐gp expression in CCA with gemcitabine resistance.

We at first tested whether expression of P‐gp was significantly elevated in gemcitabine‐resistant cell lines compared with that of the parental cell lines. Then, P‐gp expression was assessed after gemcitabine‐resistant cells were transfected with *TET1* gene and siRNA‐*TET1*, and the results showed that p‐gp protein expression significantly decreased in cells overexpressing TET1, whereas markedly higher p‐gp expression occurred with silencing TET1. In in vivo experiments, the results from the immunohistochemistry analysis showed the close inverse association between the expression of TET1 and P‐gp. These findings suggested that P‐gp was likely regulated by TET1 directly or indirectly in CCA with gemcitabine resistance, and we therefore proposed that *ABCB1* might be a functional target for the chemoresistance in CCA. P‐gp is an ATP‐binding cassette (ABC) transporter that contributes to MDR in cancer cells, by which P‐gp utilizes the energy from ATP hydrolysis to open the drug pathway through pumping substrates across the membrane.^35^ However, the understanding about how the expression of *ABCB1* was regulated in correlation with the acquisition of chemoresistance in cancer remains controversial. Reed et al provided strong evidence that changes in *ABCB1* promoter methylation were associated with drug resistance in breast tumor cells.^36^ However, other studies reported that *ABCB1* expression was altered in pancreatic cancer with drug resistance by a mechanism independent of promoter methylation.^37^

In our present study, the possible mechanisms underlying that TET1 regulated P‐gp expression in CCA with gemcitabine resistance and whether it is involved with promoter methylation were undefined. This result represents a main flaw and limitation in our study, along with the fact that very little work has been conducted on the mechanisms for the alterations of P‐gp expression associated with TET1. According to the latest studies worldwide, microRNAs (miRNAs) seemed as the most possible mediating factors. MiRNAs as a class of 17‐25 nucleotides small noncoding RNAs are involved in a variety of vital cellular processes by regulating the expression of target genes. Meanwhile, it is reported that TET1 could be regulated by multiple miRNAs.^38^ The recent research discovered that TET1 could also regulate the miRNA level by its demethylation ability, such as *miR‐29* family and *miR‐200*.^39^, ^40^ Additionally, many miRNAs were determined that could upregulate *MDR1/ABCB1 *gene expression leading to chemoresistance in cancer, such as *miR‐21* and *miR‐27a*.^37^ Therefore, miRNAs could become our research focus when we try to explore the mechanisms for overexpression of TET1 inducing a reduction of P‐gp expression.

In conclusion, our study described that overexpression of TET1 strengthened the sensitivity of CCA to gemcitabine accompanied by a decrease in P‐gp expression. Additionally, our data showed that decreased expression of TET1 was associated with poorer prognosis of CCA patients with chemotherapy, and the level of TET1 expression has a potential value for the prediction of clinical outcomes in CCA patients with chemotherapy. Thus, our study indicated that TET1 could be a promising target for overcoming chemoresistance in cancer. However, further work should be devoted to exploration of the mechanisms involved in the effects of TET1 expression on chemoresistance in CCA.
