# Supplementary figures and images for: Enhanced expression of ten‐eleven translocation 1 reverses gemcitabine resistance in cholangiocarcinoma accompanied by a reduction in P‐glycoprotein expression
Source: Cancer Med. 2019 Feb 19;8(3):990–1003. doi: 10.1002/cam4.1983 (PMC6434196; doi:10.1002/cam4.1983)

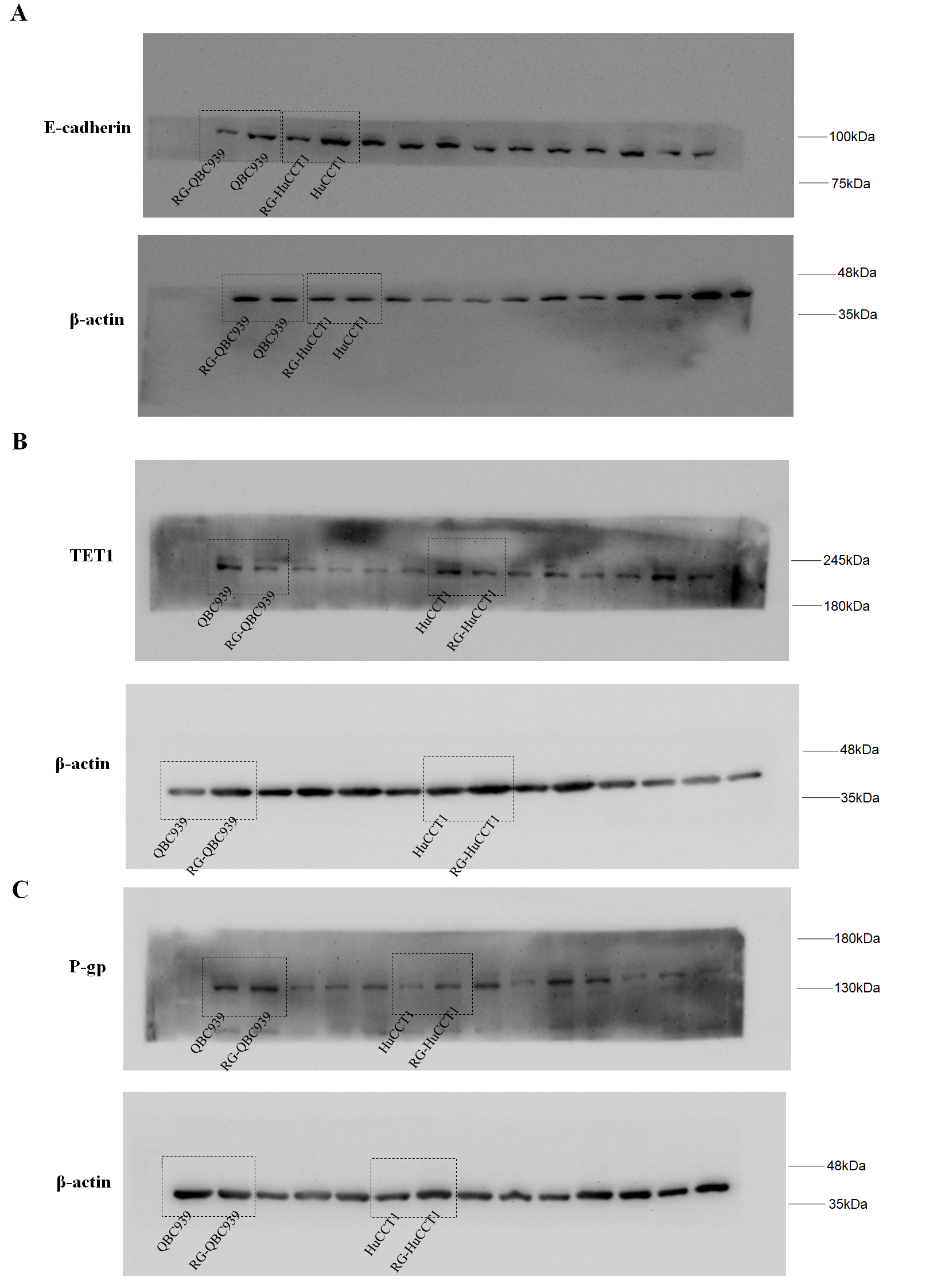

Supplement: Supplementary file 1 [file CAM4-8-990-s001.tif]

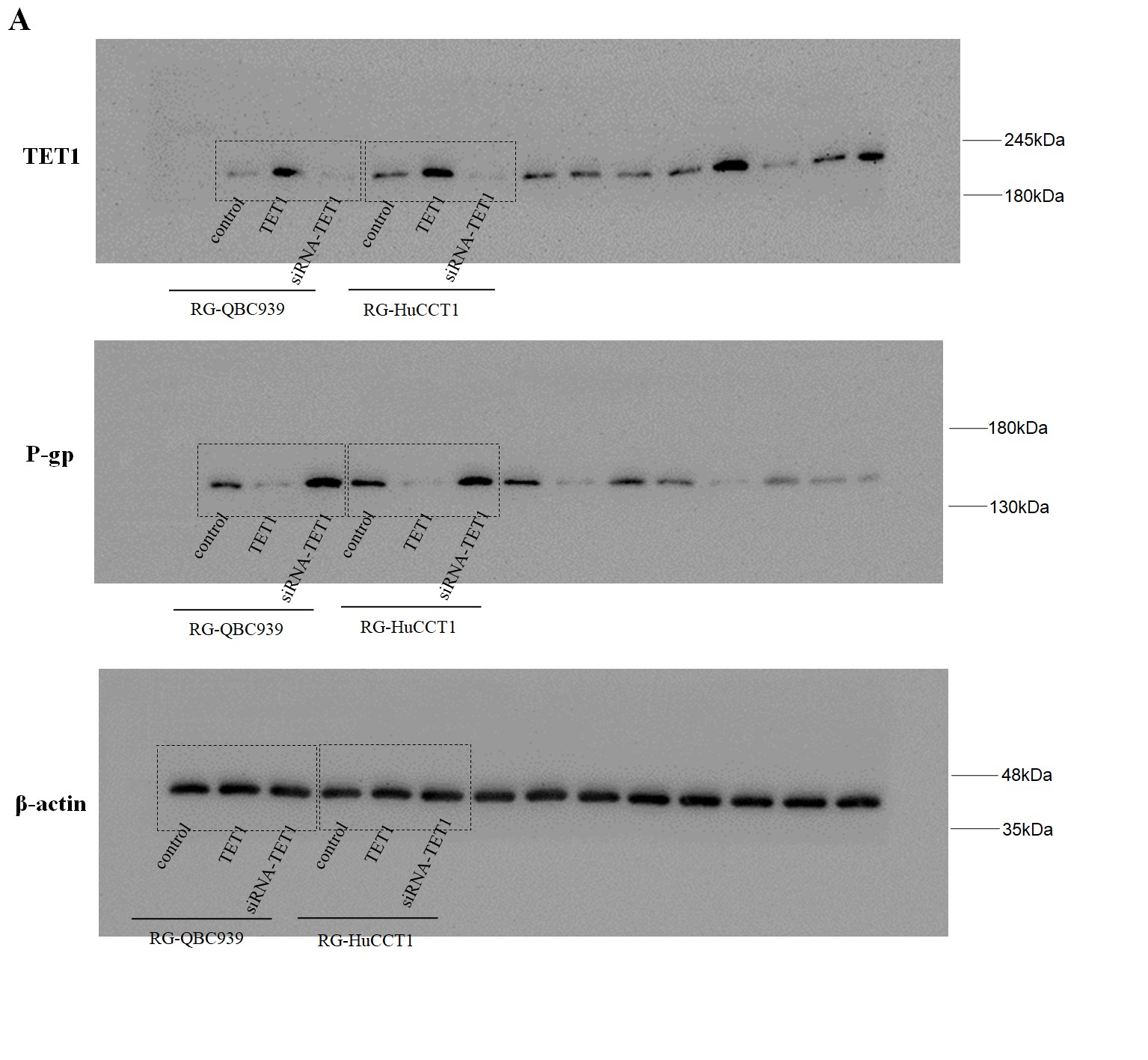

Supplement: Supplementary file 2 [file CAM4-8-990-s002.tif]
